# Multimodal Enzyme‐Carrying Suprastructures for Rapid and Sensitive Biocatalytic Cascade Reactions

**DOI:** 10.1002/advs.202104884

**Published:** 2021-12-22

**Authors:** Seong‐Min Jo, Jihye Kim, Ji Eun Lee, Frederik R. Wurm, Katharina Landfester, Sanghyuk Wooh

**Affiliations:** ^1^ Max Planck Institute for Polymer Research Ackermannweg 10 Mainz 55128 Germany; ^2^ School of Chemical Engineering & Materials Science Chung‐Ang University Heukseok‐ro 84 Dongjak‐gu Seoul 06974 Republic of Korea; ^3^ Sustainable Polymer Chemistry Group MESA+ Institute for Nanotechnology Faculty of Science and Technology Universiteit Twente PO Box 217 Enschede 7500 AE The Netherlands

**Keywords:** biocatalytic cascade reactions, colloidal suprastructures, glucose assays, liquid repellent surfaces, surface‐templated suprastructure synthesis

## Abstract

Colloidal assemblies of mesoporous suprastructures provide effective catalysis in an advantageous volume‐confined environment. However, typical fabrication methods of colloidal suprastructures are carried out under toxic or harmful conditions for unstable biomolecules, such as, biocatalytic enzymes. For this reason, biocatalytic enzymes have rarely been used with suprastructures, even though biocatalytic cascade reactions in confined environments are more efficient than in open conditions. Here, multimodal enzyme‐ and photocatalyst‐carrying superstructures with efficient cascade reactions for colorimetric glucose detection are demonstrated. The suprastructures consisting of various functional nanoparticles, including enzyme‐carrying nanoparticles, are fabricated by surface‐templated evaporation driven suprastructure synthesis on polydimethylsiloxane‐grafted surfaces at ambient conditions. For the fabrication of suprastructures, no additional chemicals and reactions are required, which allows maintaining the enzyme activities. The multimodal enzymes (glucose oxidase and peroxidase)‐carrying suprastructures exhibit rapid and highly sensitive glucose detection via two enzyme cascade reactions in confined geometry. Moreover, the combination of enzymatic and photocatalytic cascade reactions of glucose oxidase to titanium dioxide nanoparticles is successfully realized for the same assay. These results show promising abilities of multiple colloidal mixtures carrying suprastructures for effective enzymatic reactions and open a new door for advanced biological reactions and enzyme‐related works.

## Graphical abstract

Multimodal enzyme‐ and photocatalyst‐carrying suprastructures are proposed in this study based through surface‐templated evaporation driven synthesis on a liquid repellent surface. The mesoporous suprastructures efficiently assist the enzymatic‐ and photocatalytic stepwise cascade reactions by providing confined geometry, which enables to create a platform of rapid and sensitive enzymatic cascade reaction.

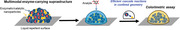

## 1 Introduction

Suprastructures are three‐dimensionally assembled structures of micro/nano colloids as building blocks.^[^ ^1^, ^2^, ^3^ ^]^ The assembled colloids as suprastructures form mesoporous structures while the intrinsic properties of the colloids are maintained. Due to the porosity with a large surface area in confined geometry, the suprastructures are useful materials in various fields, for example, templates of optics,^[^ ^4^, ^5^, ^6^ ^]^ electrodes of electrochemical cells,^[^ ^7^, ^8^ ^]^ and catalysts.^[^ ^9^, ^10^ ^]^ Typically, suprastructures are fabricated by spray drying process^[^ ^11^, ^12^ ^]^ or colloidal self‐assembly in solvents,^[^ ^3^, ^13^, ^14^, ^15^, ^16^ ^]^ which require solvents, chemicals, or heat, or both. This harsh condition of the fabrication process has been a hurdle to form suprastructures with unstable biomolecules. Recently, Wooh et al. proposed a new way to fabricate suprastructures on liquid repellent surfaces, named surface‐templated evaporation driven (S‐TED) synthesis.^[^ ^17^ ^]^ The S‐TED method can easily control the size, shape, and porosity of the suprastructures.^[^ ^18^, ^19^, ^20^ ^]^ In addition, this method does not require toxic solvents, additional heat and pressure, and pH changes during the fabrication process. Suprastructures are spontaneously formed on liquid repellent surfaces by evaporation of aqueous colloidal dispersion drops in atmospheric pressure and room temperature. Therefore, the S‐TED suprastructure synthesis is a good candidate method to apply sensitive biomolecules, such as, enzymes, into suprastructures.

Enzymes catalyze numerous biological reactions in living organisms and artificial reaction systems. Many studies have demonstrated that more efficient enzymatic cascade reactions can be carried out in a confined environment than a dispersed environment.^[^ ^21^ ^]^ For example, core/shell or mesoporous nanoparticles (NPs) are extensively explored miniature reactors to confine the enzymes and their reactions. Notwithstanding the development of such NPs for confining enzymes,^[^ ^22^, ^23^, ^24^, ^25^, ^26^ ^]^ the limited volume of the cavity in NPs remains a challenge. Suprastructure consisting of NPs can provide proximity among assembled NPs that allows a large volume of the cavity for incorporating enzymes. Therefore, in this study, we suggest multimodal enzyme‐carrying suprastructures fabricated by S‐TED method providing mesoporous confined geometries for enzymatic cascade reactions. In order to demonstrate suprastructure as an enzymatic reaction platform, suprastructures are fabricated with a dispersion mixture of two different silica NPs encapsulating glucose oxidase (GOX) and horseradish peroxidase (HRP), which are useful enzymes for a variety of fields.^[^ ^27^, ^28^, ^29^, ^30^, ^31^, ^32^, ^33^, ^34^, ^35^, ^36^, ^37^, ^38^, ^39^, ^40^ ^]^ GOX oxidizes glucose into gluconic acid and hydrogen peroxide, which has extensively been applied to develop the glucose‐, pH‐, and hydrogen peroxide‐responsive systems. HRP activates various dyes by consuming hydrogen peroxide, therefore, the combination of GOX and HRP have been used for glucose biosensors. As the cascade reaction of GOX and HRP has been studied well and both enzymes have a good stability at ambient condition, GOX and HRP are good candidates to investigate enzymatic reactions in suprastructures. The mixture dispersion drops of those enzyme‐carrying silica NPs (enzyme‐carrying NPs) are dried on polydimethylsiloxane (PDMS)‐grafted glass at ambient conditions, resulting in enzyme‐carrying suprastructures. Through the S‐TED method in “gentle” conditions without any toxic solvents, surfactants, and changes of temperature, pH, and pressure, denaturation of the enzymes can be avoided, keeping their activities during the fabrication process of suprastructures.

Suprastructure is advantageous in providing a confined environment while providing a mesoporous channel to enhance enzymes and reactants accessibility. Thus, highly improved cascade reaction kinetics and sensitivity are obtained by the enzymes‐carrying suprastructures. Moreover, photocatalytic materials, such as, TiO_2_‐NPs, also can be simply embedded in the suprastructures together with enzyme‐carrying NPs. These enzyme‐ and photocatalyst‐carrying suprastructures realize the enzymatic‐photocatalytic stepwise cascade reactions, which confirm an ability to imply multi‐functionality. Thus, here we describe the colloidal suprastructures carrying biocatalytic cascade reactions for glucose assay. Based on understanding the efficient cascade reactions in the suprastructure, we develop the miniature glucose sensors that are able to perform the glucose assay using human serum and urine.

## 2 Results and Discussion

### 2.1 Fabrication of Enzyme‐Carrying Suprastructures by Surface‐Template Evaporation Driven Synthesis

**Figure 1:**
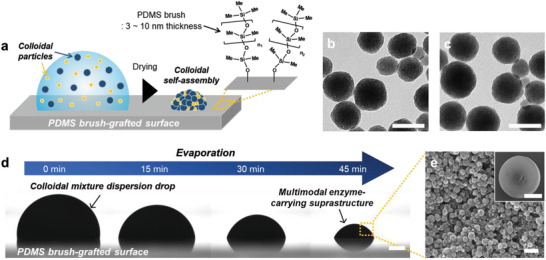
Enzyme‐carrying suprastructures of nanoparticle fabricated by a S‐TED method. a) Schematics for the fabrication of GOX‐ and HRP‐carrying suprastructures on the PDMS‐grafted surface by evaporation. TEM images of silica‐NPs carrying b) GOX and c) HRP to prepare the suprastructure. Scale bars represent 200 nm. d) Evaporation progress for the preparation of the suprastructures on the PDMS‐grafted surface (scale bar: 500 µm). e) SEM images of the suprastructure. Scale bars represent 200 nm for a high magnification image and 500 µm for a low magnification.

The hemispherical‐shaped suprastructures were fabricated on the PDMS‐grafted surfaces by drying the drops of a colloidal aqueous dispersion (**Figure** **1**a). To prepare the PDMS‐grafted glass, cleaned glass by sonication in ethanol and acetone and activated by air plasma, was incubated in a chamber filled with silicone oil (trimethylsiloxy‐terminated PDMS) at 100 °C for 1 day. During the incubation, the siloxane backbone of the silicone oil is partially cleaved and connected to hydroxyl groups of the plasma‐treated glass surfaces resulting in PDMS grafting.^[^ ^41^ ^]^ The PDMS‐grafted surface exhibits good liquid repellency for both polar and non‐polar liquid drops due to the flexible siloxane bond of the PDMS chain. The low contact angle hysteresis (Δ*θ* = 7 ± 0.3°) and sliding angle (*θ*_SL_ = 14 ± 0.7° for 5 µL drop) of water drops confirmed good water repellency with fast depinning of contact line on the PDMS‐grafted surface (Table S1, Supporting Information).^[^ ^41^, ^42^, ^43^, ^44^, ^45^ ^]^

The dispersion drops were prepared by mixing two as‐synthesized enzymes (GOX or HRP)‐carrying NP dispersions. These enzyme‐carrying silica NPs were synthesized by a sol‐gel reaction in microemulsion with covalently bound enzymes to the silica matrix.^[^ ^46^ ^]^ Both enzyme‐carrying silica NPs (GOX‐ and HRP‐NPs) exhibited 100–200 nm in diameter (Figure 1b,c).^[^ ^46^ ^]^ The enzymatic activities of each enzyme‐carrying NP (GOX for 15% and HRP for 10% compared to the native enzyme) were reported previously.^[^ ^46^ ^]^ Detailed characteristics of the NPs about diameter, zeta potential, pore volume, and density are described in Table S2, Supporting Information. In particular, we found that the hydrodynamic diameters of individual GOX‐NPs, HRP‐NPs, and the mixture of two NPs were almost the same, which verified good dispersity of NPs with no agglomeration by mixing (Figure S1, Supporting Information).

The dispersion drops (5 µL) containing GOX‐ and HRP‐carrying NPs with a volume ratio of 1:2 (60 mg mL^−1^ each) were dropped onto the PDMS‐grafted surface. As the size of both GOX‐ and HRP‐NPs are nearly identical, the drops contain 1:2 number ratio of GOX‐NPs:HRP‐NPs. Then, evaporation of the dispersion drops led to the shrinking of their volume. Owing to the water repellency of the PDMS‐grafted surface, the contact lines of the dispersion drops moved to the center by volume shrinkage (Figure 1d). After the complete evaporation of the water (≈45 min), the successful fabrication of hemispherical suprastructures consisting of a densely packed co‐assembly of the GOX‐and HRP‐carrying NPs was achieved (Figure 1e). The size of the suprastructures can be easily controlled in the range from microns to millimeters by tuning the concentration and volume of the NP drops. In this study, the hemispherical suprastructures with millimeter‐scale size (diameter = 1.72 mm and height = 0.52 mm, averaged by 40 suprastructures) were chosen for colorimetric glucose assay (Figure S2, Supporting Information). For comparison, we also prepared hemispherical suprastructures by using silica NPs without enzymes. The suprastructure with no enzymes exhibited similar morphology with enzyme‐carrying suprastructures, confirming that the enzymes encapsulated in the silica NPs rarely influenced on constructing the structure (Figure S3, Supporting Information). In addition, we fabricated the suprastructure with commercially available silica NPs and free enzymes, GOX, and HRP (Figure S4, Supporting Information). In this case, the amount of enzyme that can be loaded into the superstructure is limited because the free enzymes decrease the surface tension of the dispersion drop which results in cracked suprastructure. The hemispherical suprastructures from the commercial silica NPs and free enzymes were formed only when the drop included fewer amounts of enzymes a factor of 0.2 than the suprastructure consisting of the enzyme‐encapsulated NPs. Therefore, for an efficient enzymatic reaction, it is certainly necessary to apply the enzyme‐encapsulated NPs as a primary particle of the suprastructure.

Suprastructure is a highly porous structure assembled with primary particles. Porosity, a ratio of pore volume and suprastructure volume, is important for suprastructures because it influences in reaction characteristics inside the suprastructure as well as mechanical property. The porosity of the suprastructure can be estimated by comparing the volume of NPs in dispersion and the volume of suprastructure, as equation below. From the equation with the volumes of dispersion (0.08 µL) and suprastructure (0.26 µL), the porosity of hemispherical suprastructure is calculated as 68 ± 0.7%.

(1) $$\begin{array}{c} \begin{array}{c} \mathrm{Porosity}\left(\%\right)=\frac{\left(\mathrm{Volume}\;\mathrm{of}\;\mathrm{the}\;\mathrm{suprastructure}\right)-\left(\mathrm{Volume}\;\mathrm{of}\;\mathrm{NPs}\right)}{\left(\mathrm{Volume}\;\mathrm{of}\;\mathrm{the}\;\mathrm{suprastructure}\right)}\times 100 \end{array} \end{array}$$

The S‐TED method allows fabricating suprastructures of different shapes by controlling the initial contact angle and/or contact line friction of drops.^[^ ^47^ ^]^ The shape of the suprastructure does not affect the porosity. Even though the shapes are different, when the suprastructures are fabricated using the same dispersion, the porosity of the suprastructures is the same. We confirmed the spherical suprastructure prepared on the soot‐templated superamphiphobic surface from the aqueous enzyme‐carrying NPs dispersion drop. The porosity of the spherical suprastructure (≈67%) is almost the same as that of the hemispherical suprastructure (≈68%) (Figure S5, Supporting Information). In fact, suprastructures of other shapes, such as spherical shape, can be applied in applications of enzymatic cascade reactions. In this study, however, we mostly used hemispherical shape for the enzymatic cascade reaction measurements because the flat‐bottomed hemispherical suprastructure has advantages of easy handling and observation of the reaction process.

### 2.2 Enzymatic Cascade Reactions of Glucose Oxidase/Horseradish Peroxidase in Suprastructure

**Figure 2:**
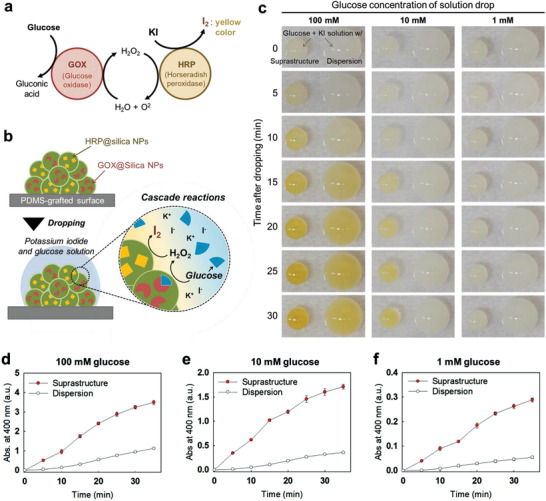
GOX/HRP cascade reactions of the suprastructure at millimolar glucose levels. a) Enzymatic reactions and signal generation principle of glucose assay using KI as a chromogen. b) Schematics for the suprastructure‐based glucose assay. c) Pictures of glucose assay for 100, 10, and 1 mm concentration on suprastructures (smaller drops on left side) and non‐dried NP dispersions (larger drops on right side) after different reaction times. The yellow‐brown color indicates the progression of the cascade reactions. d–f) Reaction progression respective to (c) through nanodrop spectroscopy (absorbance at 400 nm). All measurements were conducted three times to get average values.

The enzymatic cascade reactions with GOX and HRP, typical reactions for the colorimetric glucose assay, are described in **Figure** **2**a. GOX catalyzes the conversion of glucose and oxygen (O_2_) into gluconic acid and hydrogen peroxide (H_2_O_2_). Then, HRP consumes the hydrogen peroxide to covert potassium iodide (KI) to iodine (I_2_), resulting in yellow‐brown color and absorbance at 250–450 nm of solution (Figure S6, Supporting Information). Therefore, the cascade reactions cause a color change of the reactant solution.^[^ ^48^ ^]^ Higher glucose concentrations result in a darker color after the reaction. This iodine‐based colorimetric scheme is widely used in commercial glucose assay strips. As this assay is based on colorimetric readout by naked eyes, point‐of‐care testing (POCT) of glucose without measuring instruments is possible. Here, we installed the enzymatic cascade reaction in the enzyme‐carrying suprastructures, and demonstrate the colorimetric glucose assay. To initiate the assay, a solution of glucose (as an analyte) and potassium iodide (as an indicator) was dropped onto the suprastructures consisting of GOX‐and HRP‐carrying NPs (Figure 2b). The aqueous solution is quickly absorbed into the suprastructure, which leads to the cascade reaction of GOX to HRP inside the confined space of the suprastructure.

Glucose (1, 10, and 100 mm) and KI (30 mm) mixture solution (glucose/KI solution) drops of 1.2 µL were placed onto the suprastructures (left drops of Figure 2c). Typically, the enzyme cascade reactions are carried out by mixing analyte, indicator, and enzyme solutions. Therefore, the enzyme‐carrying NPs aqueous dispersion drops (5 µL) were used as a control, which contains the same amount of GOX‐ and HRP‐carrying NPs with the suprastructure. In this case, the glucose/KI solutions (1.2 µL) were added to the enzyme‐carrying NPs dispersion drops (5 µL) for the assay (right drops of Figure 2c). Color changes of 100 mm glucose solution drops can be recognized in 5 and 15 min after dropping on the suprastructure and the dispersion drop, respectively. 30 min after dropping, the glucose solution drops on both suprastructure, and dispersion drop showed yellow‐brown color, the color on the suprastructure was darker than that on the dispersion. A more significant difference of color change was observed for 10 mm glucose solution between the suprastructure and NP dispersion. The yellow color appeared in 15 min after dropping and gradually became darker on the suprastructure, while no color appeared on the NP dispersion drop even 30 min after dropping. From photographic pictures, we verified decreases in the blue element among the RGB (red, green, and blue) color elements (Figure S7, Supporting Information). Remarkably, the progression of reactions in suprastructure seems to be much faster than reactions in NP dispersion. The concentration of 1 mm glucose, does not lead to a significant color change by simple eye inspection on both suprastructure and NP dispersion drop in 30 min.

For precise detection and quantification, the Nanodrop spectroscopy measuring the absorbance of microliter solution drops was performed (Figure 2d–f). The production of I_2_ via the cascade reaction can be confirmed by measuring the absorbance at 400 nm. Remarkably, meaningful increases in absorbance are detected by the Nanodrop spectroscopy for the 1 mm glucose solution (Figure 2f). In addition, the enzymatic cascade reactions of all the concentrations of glucose solutions in superstructures were ≈5 to 7 times faster than the reactions in NP dispersions for 30 min reaction time. In fact, dropping after 30 min, an effect of evaporation on color change becomes strong (evaporation becomes faster as volume is shrunken), and the drying is completed in 35–40 min. Therefore, a standard curve from the absorbance of 400 nm at 20 min reaction time was prepared, which exhibits accurate comparison of enzymatic activities of the NPs dispersion and the suprastructure (Figure S8, Supporting Information).

In addition, we found that this rapid cascade reaction of the suprastructure is reusable by washing the remaining reactants and products with water. The glucose solution dropped onto the washed suprastructure after the first reaction showed the color change to yellow‐brown again, indicating a successful enzymatic cascade reaction (Figure S9, Supporting Information). It confirms that the enzymes are strongly fixed in the suprastructure keeping their catalytic properties during the reaction, which allows the suprastructure to develop a sustainable assay system.

### 2.3 Enzymatic Kinetics of Cascade Reactions in Suprastructural Confinement

**Figure 3:**
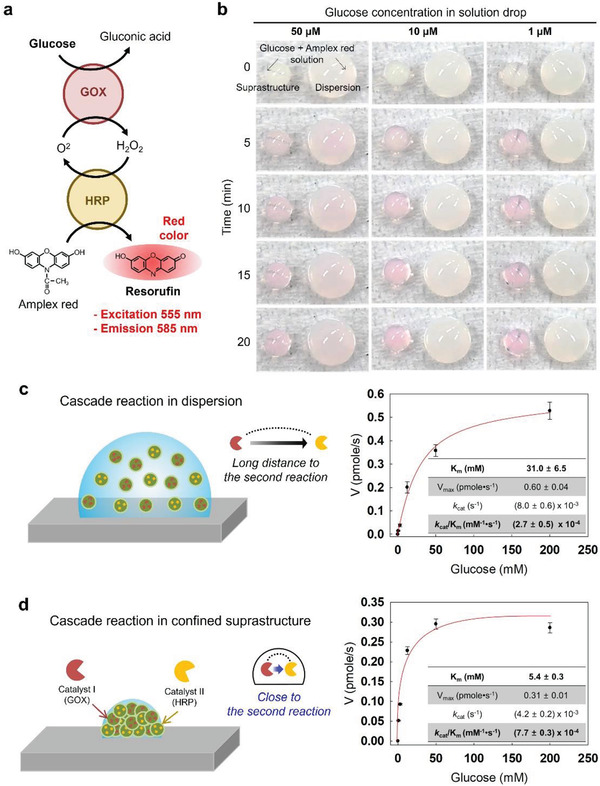
Reaction kinetics of GOX/HRP cascade reactions. a) Enzymatic reactions and signal generation principle of glucose assay using Amplex red dye. b) GOX/HRP cascade reactions at micromolar glucose levels. Pictures of glucose assay for 50, 10, and 1 µM solution drops on suprastructures (left drops) and non‐dried NP dispersions (right drops) in time relapse. The red color indicates the progression of the cascade reactions. c) Schematics of GOX/HRP cascade reactions in dispersion (left) and Michaelis‐Menten kinetics in terms of GOX (right). d) Schematics of GOX/HRP cascade reactions in suprastructure (left) and Michaelis‐Menten kinetics in terms of GOX (right).

**Figure 4:**
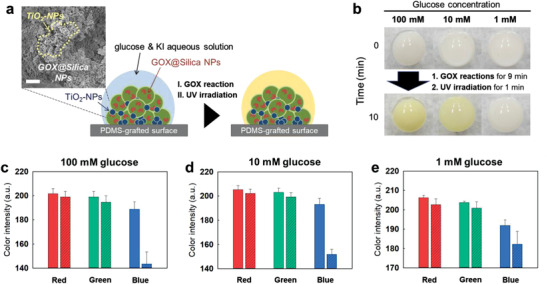
GOX/TiO_2_ cascade reactions on suprastructure. a) Schematics of GOX‐ and TiO_2_‐carrying suprastructure‐based glucose assay, and SEM image of the cross‐sectioned GOX‐TiO_2_ suprastructure (scale bar: 1 µm). b) Photographic pictures of glucose assay on suprastructures for 100, 10, and 1 mm solution drops. The yellow‐brown color indicates the progression of the reactions. The GOX reactions were allowed for 9 min, then, UV‐A was irradiated for 1 min to allow TiO_2_ reactions. c–e) Color intensities of solutions after finishing the cascade reactions, characterized by image analysis of pictures (the left plain bars: Before reactions; the right diagonal‐checked bars: After reactions). Each result was averaged from 3 samples.

In order to monitor the cascade reaction at lower concentrations of less than 1 mm glucose, we changed the indicator from KI to Amplex red, which allows more sensitive glucose detection than KI. Amplex red (no color) can be converted to resorufin (red color) by the GOX/HRP cascade reaction (**Figure** **3**a). In the Amplex red dye system, enzyme‐carrying suprastructures also exhibited a rapid cascade reaction with high sensitivity. The suprastructures with Amplex red dye were able to detect glucose concentrations down to 1 µM by the naked eyes (left drops in **Figure** **4**b). In contrast, the use of NP dispersion with the same dye did not allow to determine glucose lower than 50 µM by naked eyes (right drops in Figure 3b).

We investigated Michaelis‐Menten reaction kinetics, including catalytic efficiency of the suprastructure and the NPs dispersion in terms of GOX/HRP cascade reaction (Figure 3c,d).^[^ ^49^ ^]^ We monitored the initial velocity of the reactions (*V*) in different glucose concentrations, then calculated the affinity of substrates (*K*_m_), turnover number (*k*_cat_), and catalytic efficiency (*k*_cat_/*K*_m_) (Figure S10, Supporting Information). It was calculated that the *k*_cat_/*K*_m_ of the suprastructure (7.7 × 10^–4^ mm ^–1^ S^–1^) is higher than that of the NP dispersion (2.7 × 10^–4^ mm ^–1^ S^–1^) by a factor of 2.9. In particular, the *K*_m_ of the suprastructure (5.4 mm) is remarkably lower than that of the NPs dispersion (31 mm), which mainly contributes the catalytic efficiency enhancement of the suprastructure. (**Note**: Low *K*_m_ indicates the high affinity between enzymes and substrates). This efficient cascade reaction in the suprastructure is mainly attributed to the proximity of two enzymes in “micro‐confined” environments (scheme in Figure 3d). Previous reports demonstrated that the crowed‐effects in the volume‐confined space could increase the efficiency of enzymatic reactions.^[^ ^21^ ^]^ The surface‐templated evaporation‐driven (S‐TED) method allows to fabricate the micro‐confined enzyme‐carrying suprastructures preserving the activities of the enzymes (Figure S11, Supporting Information). The S‐TED fabrication process is carried out in ambient conditions avoiding solvents, surfactants, high temperature, and chemical reactions. Therefore, the enzymatic activities of single NPs are not changed after the fabrication. Only the NPs are confined as a suprastructure.

In addition, when the glucose solution was added to the suprastructures, there is no dilution effect for the sample. For a classical assay, an analyte solution (e.g., glucose) is added into an assay solution (e.g., KI, enzyme solution), resulting in a dilution of analytes, indicators, and enzymes, decreasing reaction speed and sensitivity. Using a highly concentrated indicator or assay on a confined hydrophobic pillar structure can reduce this issue.^[^ ^30^ ^]^ However, it cannot perfectly solve the issue because the analyte solution is still diluted. Unlike the enzyme‐carrying NP dispersion, the dried enzyme‐carrying suprastructures completely avoid this dilution issue, allowing rapid and highly sensitive enzymatic cascade reactions.

### 2.4 Effect of Size and Porosity of Suprastructures on Reaction Kinetics

By the S‐TED method, the size of the suprastructure can be easily tuned by changing the concentration or volume of the initial dispersion drops. In addition to the hemispherical suprastructure of 1.7 mm diameter, we prepared suprastructures of various sizes from 1.5 to 3 mm in diameter by using dispersion drops of different volumes (Figure S12, Supporting Information). Regarding the reaction kinetics, larger suprastructure exhibited faster reaction velocity (*V*) because larger suprastructure had larger amounts of the enzyme‐carrying NPs. Therefore, size control is one of the simplest ways to regulate the reaction kinetics of the suprastructure cascade reaction system.

In addition, we showed an effect of porosity on reaction kinetics. The hemispherical suprastructure of higher porosity was prepared by drying an Ouzo containing NPs dispersion drop (Figure S13, Supporting Information). For the Ouzo dispersion drop, *trans*‐anethole oil and ethanol were added in the enzyme‐carrying NPs dispersion.^[^ ^20^ ^]^ Microdroplets of *trans*‐anethole oil dispersed in the NPs dispersion drop formed micropores after finishing the evaporation, which increased the porosity up to 75%. Higher porosity is more favorable for the diffusion of the reactants. Therefore, the suprastructure of 75% porosity exhibited 1.8‐times higher catalytic efficiency (*k*_cat_/*K*_m_: 1.4 × 10^–3 ^mm ^–1^ S^–1^) than that of 68% porosity (*k*_cat_/*K*_m_: 7.7 × 10^–43 ^mm ^–1^ S^–1^). However, highly porous suprastructures also leads low mechanical strength.^[^ ^18^ ^]^ Optimal porosity should be considered when the suprastructure is applied to assay applications.

### 2.5 Enzymatic‐Photocatalytic Heterogeneous Cascade Reactions of Enzyme‐Photocatalyst in Suprastructures

In addition to enzymatic reactions, suprastructures fabricated by the S‐TED method can have various functions by incorporating different functional colloids. For example, a photocatalytic activity was incorporated with an enzymatic reaction in a single suprastructure, realized by co‐assembly of GOX‐carrying NPs and titanium dioxide (TiO_2_). The GOX‐ and TiO_2_‐carrying suprastructure was fabricated on the PDMS‐grafted surface by drying the dispersion drop (5 µL) of a mixture of GOX‐carrying NPs and TiO_2_‐NPs (diameter: ≈30 nm) with a volume ratio of 9:1 (Figure S14, Supporting Information). The co‐assembled GOX NPs (diameter: 100–200 nm) and TiO_2_‐NPs (diameter: ≈30 nm) in a single suprastructure were confirmed by scanning electron microscopy (Figure 4a and Figure S15, Supporting Information).

To demonstrate the enzymatic‐photocatalytic cascade reactions, the glucose (1, 10, and 100 mm) and KI (30 mm) mixed solution (1.2 µL) was dropped onto the suprastructure. The glucose reacted with GOX in the suprastructure for 9 min at ambient conditions. Then, UV‐A light (wavelength: 310–400 nm) was irradiated for 1 min; color changes to yellow/brown were immediately detected by naked‐eye at the 10 and 100 mm glucose drops (Figure 4b). In addition, the decreases in the blue elements of the drops in all samples (1, 10, and 100 mm glucose) were precisely analyzed by image analysis of the pictures (Figure 4c–e). It indicates that the stepwise heterogeneous cascade reaction using GOX and TiO_2_ as catalytic elements was successfully carried out. First, hydrogen peroxide is produced by the GOX reaction. Then, KI (I^–^) is oxidized into I_2_ by photocatalytic activity of TiO_2_ in the presence of hydrogen peroxide; the TiO_2_ photocatalyst substitutes peroxidase (see the equations below). The effect of TiO_2_ was verified with the suprastructures of various TiO_2_ contents. Unfortunately, homogeneously mixed suprastructures with TiO_2_ ratio above 10 wt% were difficult to be obtained because TiO_2_ NPs aggregated in dispersion. For the suprastructures including 2.5–10 wt% TiO_2_, higher amount of TiO_2_ caused faster reaction, characterized by color changes (Figure S16, Supporting Information). This result clearly indicates that the TiO_2_ activates KI with hydrogen peroxide, generated by the GOX‐glucose reaction, to I_2_ in the suprastructure though the optimum content of TiO_2_ is higher than 10 wt%. The peroxidase‐like activity of TiO_2_ has also been reported in previous reports.^[^ ^50^, ^51^ ^]^ The possible chemical pathway of iodide (I^–^) to iodine (I_2_) conversion by the TiO_2_ peroxidase‐like reaction can be presumed based on the activity of photocatalyst and natural peroxidase.^[^ ^52^, ^53^, ^54^ ^]^ It is well known that the photocatalytic activity of TiO_2_ produces proton and hydroxyl radicals in water/hydrogen peroxide mixture by generating holes and electrons. Then, the produced proton helps the oxidization of iodide into iodine in the presence of hydrogen peroxide, as described in Equation (1).^[^ ^52^ ^]^

(2) $$H_{2}O_{2}+2I^{-}+2H^{+}\;\to \;I_{2}+2H_{2}O$$

In addition, the iodide ion acts as a scavenger of the hydroxyl radical and becomes an iodine ion that also induces the formation of iodine, as described in Equations (2) and (3).^[^ ^55^, ^56^ ^]^

(3) $$I^{-}+HO^{•}\to I^{•}+OH^{-}$$

(4) $$I^{•}+\;I^{-}\to I_{2}^{•-}\to I_{2}+e^{-}$$

The sensitivity for the assay on the GOX/TiO_2_‐carrying suprastructures is similar to that on the GOX/HRP‐carrying suprastructures. This high efficiency of the heterogeneous cascade reactions is also due to the proximity of two catalysts, GOX and TiO_2_, in a confined suprastructure. This achievement shows the versatility of the suprastructure fabricated by the S‐TED method for multimodal biocatalytic reactions.

### 2.6 Glucose Assay in Human Urine and Serum

**Figure 5:**
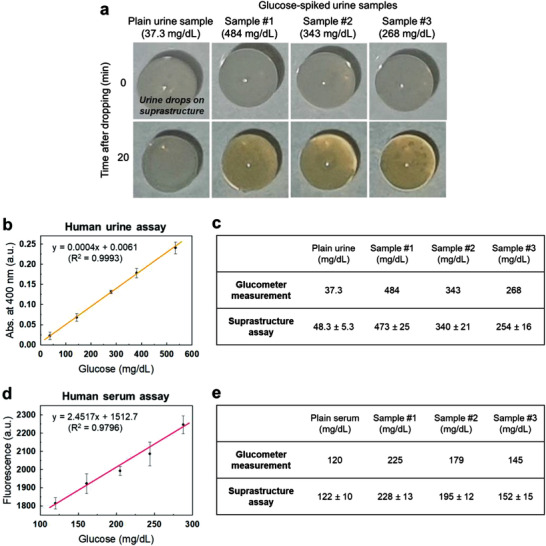
Glucose assay in human urine and serum through a GOX/HRP cascade reactions. a) Pictures of glucose assay for human urine samples. The generation of yellow‐brown color after 20 min indicates the progression of the reactions. b) A standard curve of GOX/HRP cascade reactions for respective glucose concentrations in human urine. KI was used as the indicator. The absorbance was measured at 400 nm. c) Comparison of urine glucose quantification by assay of suprastructures and a commercial glucometer. d) A standard curve of GOX/HRP cascade reactions for respective glucose concentrations in human serum. Amplex red was used as the indicator. Fluorescence was obtained at excitation 555 nm and emission 595 nm. e) Comparison of urine glucose quantification by assay of suprastructures and a commercial glucometer. (Note: Unit conversion for glucose concentration; 37.3 mg dL^−1^ (2.1 mm), 484 mg dL^−1^ (27 mm), 343 mg dL^−1^ (19 mm), 268 mg dL^−1^ (15 mm), 120 mg dL^−1^ (6.7 mm), 225 mg dL^−1^ (12.5 mm), 179 mg dL^−1^ (9.9 mm), 145 mg dL^−1^ (8.1 mm)).

The enzyme‐carrying suprastructure can potentially be applied for enzyme‐based diagnostics such as the detection of glucose in human urine. Urine is a simple and useful specimen to detect several malfunctions or diseases, for example, Diabetes Mellitus.^[^ ^57^, ^58^ ^]^ We used pooled‐urine as a sample. The glucose concentration of the human urine was determined by a commercial glucometer. Additional glucose was spiked into the plain urine sample to constitute the diabetic level of the blood sugar. Then, the mixture of the urine and KI solution (9:1 volume ratio; 30 mm KI) was dropped onto the GOX/HRP‐carrying suprastructures to demonstrate a glucose assay through enzymatic cascade reactions. We found the generation of the yellow‐brown color at glucose‐spiked urine samples that are the diabetic levels of glucose (#1: 484 mg dL^−1^, #2: 343 mg dL^−1^, and #3: 268 mg dL^−1^, respectively) (**Figure** **5**a). In contrast, almost no changes in color were observed by naked eyes on the plain urine samples in 20 min, which are in the range of normal glucose level (37.3 mg dL^−1^). We achieved the suprastructure‐based glucose assay using urine samples, which enables naked eyes to read the changing color in diagnostic levels. Normally, fresh urine samples contain inhibitors or non‐buffered conditions, leading to slower enzymatic reactions than ideal buffered conditions. Due to the favorable reaction conditions of the confined environments, the enzymatic cascade reactions in the suprastructures were not significantly inhibited in urinary conditions.

We found that the absorbance (at 400 nm) of urine samples, measured by the nanodrop spectroscopy, was proportional to the glucose concentration. Therefore, by monitoring the relation of absorbance and concentration, a standard curve of changes in absorbance with defined glucose concentration could be realized, which allows an estimation of glucose concentration of unknown samples (Figure 5b). We obtained comparable results between the values from the suprastructure‐based assay and the commercial glucometer (Figure 5c). By using the suprastructure platforms, precise quantification of glucose could be achieved at diagnostic levels of the urine sample (±5% difference with a glucometer). Given its simple and accurate application, the suprastructure is expected to be a promising platform for biosensors.

Moreover, we also performed the glucose assay for human serum. The glucose concentration of the human serum was determined by the glucometer. Amplex red was used for the indicator that allows glucose assay by measuring fluorescence (excitation: 555 nm, emission: 595 nm) of the serum reactant. At first, different amount of glucose was spiked into plain serum (120 mg dL^−1^), and glucose assay was performed with Amplex red to obtain a standard curve in the range of 100–300 mg dL^−1^ (Figure 5d). Then, we prepared three randomized serum samples that simulated the diabetic levels of blood sugar (#1: 225 mg dL^−1^, #2: 179 mg dL^−1^, and #3: 145 mg dL^−1^, respectively). Using the serum, we successfully achieved the glucose quantification by the suprastructure‐based assay that showed comparable results to the value of the glucometer (Figure 5e). Efficient enzymatic reactions in body fluids are of great challenges due to harsh conditions by the presence of proteolytic factors, high viscosity, and adsorption of serum proteins. Our results for efficient enzyme reactions in serum imply that the suprastructures might be applied for implantable and injectable bio‐devices in vivo environments.

## 3 Conclusions

In summary, enzymatic catalysts in confined suprastructure exhibit the enhanced cascade reaction kinetics due to increased proximity between two catalysts. Our current work introduces the first example of multimodal enzyme‐carrying suprastructures through the S‐TED synthesis with various functional colloids (e.g., TiO_2_‐NPs, GOX‐ and HRP‐carrying NPs). This method enables to fabrication of suprastructure in a tailorable fashion without toxic solvents, chemicals, and heat, preserving activities of biomaterials. The enzyme (GOX) and photocatalyst (TiO_2_) co‐carrying suprastructures also easily achieve the enzyme to photocatalyst heterogeneous cascade reactions. Significantly, rapid and highly sensitive cascade reactions for glucose assay are represented on all the suprastructures via reactions in confined geometry and non‐dilution effect of the solution. Therefore, we anticipate that the future development of suprastructures with various biomaterials will play an important role in designing multimodal biomaterials. Moreover, suprastructures elucidate their structural and processible benefits for efficient, practical POCT diagnostic tools (e.g., glucose assay using human urine or bloods). Beyond in vitro purposes, the suprastructures have a potential for bioreaction‐based implantable and injectable bio‐devices such as, drug carriers, body sensors, and tissue‐engineered organs.

## Conflict of Interest

The authors declare no conflict of interest.

## Supporting information

Supporting Information

## Data Availability

Research data are not shared.
