# Multimodal Enzyme‐Carrying Suprastructures for Rapid and Sensitive Biocatalytic Cascade Reactions

**DOI:** 10.1002/advs.202204665

**Published:** 2022-09-23

**Authors:** Seong‐Min Jo, Jihye Kim, Ji Eun Lee, Frederik R. Wurm, Katharina Landfester, Sanghyuk Wooh

*Adv. Sci*. **2021**, *9*, 2104884

DOI: 10.1002/advs.202104884

In the subpart “2.5. Enzymatic‐Photocatalytic Heterogeneous Cascade Reactions of Enzyme‐Photocatalyst in Suprastructures” of the original published article, the citation of the Equation are wrongly displayed. The corrected part is shown below:

Then, the produced proton helps the oxidization of iodide into iodine in the presence of hydrogen peroxide, as described in

Equation (2).^[52]^

(2) $$H_{2}O_{2}+2I^{-}+2H^{+}\to I_{2}+2H_{2}O$$

In addition, the iodide ion acts as a scavenger of the hydroxyl radical and becomes an iodine ion that also induces the formation of iodine, as described in Equations (3) and (4).^[55,56]^

(3) $$I^{-}+{\mathrm{HO}}^{•}\to I^{•}+{\mathrm{OH}}^{-}$$

(4) $$I^{•}+I^{-}\to I_{2}^{•-}\to I_{2}+e^{-}$$

The authors apologize for any inconvenience caused.

