# Supplementary material for: Multimodal Enzyme‐Carrying Suprastructures for Rapid and Sensitive Biocatalytic Cascade Reactions
Source: Adv Sci (Weinh). 2021 Dec 22;9(10):2104884. doi: 10.1002/advs.202104884 (PMC8981434; doi:10.1002/advs.202104884)
Supplement: Supplementary file 1 — Supporting Information [file ADVS-9-2104884-s001.pdf]

ADVANCED SCIENCE
Open Access

Supporting Information

for *Adv. Sci.*, DOI 10.1002/advs.202104884

Multimodal Enzyme-Carrying Suprastructures for Rapid and Sensitive Biocatalytic Cascade Reactions

Seong-Min Jo, Jihye Kim, Ji Eun Lee, Frederik R. Wurm, Katharina Landfester and Sanghyuk Wooh**

ADVANCED SCIENCE
Open Access

Supporting Information

for *Adv. Sci.,* DOI: 10.1002/advs. 202104884

Multimodal Enzyme-Carrying Suprastructures for Rapid and Sensitive Biocatalytic Cascade Reactions

Seong-Min Jo, Jihye Kim, Ji Eun Lee, Frederik R. Wurm, Katharina Landfester, Sanghyuk Wooh**

Materials and Methods

Materials

Glucose oxidase from *Aspergillus niger* Type X-S (GOX; EC. 1.1.3.4), D-(+)-glucose (mixture of α and β anomers), potassium iodide (KI), Amplex red (Ampliflu™ Red), tetraethyl orthosilicate (TEOS), 3-aminopropyl trimethoxysilane (APTMS), *N*-hydroxysuccinimide (NHS), triton X-100, cyclohexane, and olive oil were purchased from Sigma-Aldrich (St. Louis, MO, USA). Ammonia solution (25%; 3 M), ethanol, and 1-ethyl-3-(3-dimethylaminopropyl)carbodiimide (EDC) were obtained from Carl Roth (Karlsruhe, Germany). Horseradish peroxidase (HRP; EC. 1.11.1.7) was provided by Thermo Fisher Scientific (Waltham, Massachusetts, USA). Silicone oil (trimethylsiloxy terminated polydimethylsiloxane (PDMS), MW = 5,970) was purchased from Gelest Inc (Pennsylvania, United States). Titanium dioxide (TiO_2) nanoparticles were purchased from EVONIK (Essen, Germany). Acetone, toluene, n-hexane and tetrahydrofuran were from Samchun Chemical (Seoul, Korea). Glass substrates ($76 \times 26 \times 1$ mm^3) were provided by Paul Marienfeld GmbH (Lauda-Königshofen ,Germany). All other reagents were in reagent grade. Pooled human urine was purchased from Innovative Research, Inc (Novi, MI, Unite States).

Preparation of PDMS-grafted surface

Glass substrate was firstly cleaned with a sufficient amount of acetone under ultrasonication for 10 min. The cleaned glass was treated by oxygen plasma for 2 min to activate hydroxyl groups on the surface (Harrick, 18W). This activated glass was immediately immersed in neat silicone oil (MW = 5,970, viscosity = 100 cSt) contained in a jar. For the PDMS grafting reactions, the jar including glass substrate and silicone oil was put into an oven and heated for 24 h at 100 ℃. The resulting glass was then rinsed sequentially with n-hexane, toluene, and tetrahydrofuran to remove unreacted silicone oil.

Characterization of PDMS-grafted surface

The static contact angle, advancing contact angle and receding contact angle with water and GOX-HRP solution were examined by the contact angle measurement device (KRÜSS, Germany). Advancing angle was measured through the largest contact angle when 15 μL of water was injected using a needle fixing a 10 μL liquid drop on a PDMS-grafted substrate. Receding angle was measured through the smallest contact angle when 15 μL of water was sucked again through the needle. Contact angle hysteresis was measured through the difference between advancing and receding angles. Contact angle measurements were carried out 5 times for each sample, and then measured values were averaged.

Preparation of enzyme-carrying silica nanoparticles (enzyme-carrying NPs)

For the enzyme-carrying silica nanoparticles synthesis, water-in-oil microemulsion was prepared from a mixture of cyclohexane, 1-hexanol, triton X-100, and TEOS as a continuous phase (total 2 mL), and NHS, EDC, APTMS, enzymes (GOX for 0.4 mg or HRP for 0.2 mg) in sodium phosphate buffer (total 0.3 mL, 3 mM, pH 7.4) as an aqueous phase, under magnetic stirring at 500 rpm and room temperature.[S1] The coupling reaction was allowed to proceed for 1 h. Then, 25% ammonia solution was added to the emulsion in order to initiate hydrolysis and condensation reactions at ambient

temperature. After stirring for 24 h, the resulting dispersion was precipitated by adding ethanol of 10 mL, centrifuged at 13,000 rpm for 15 min. The precipitates were redispersed in deionized water, and dialyzed for 2 days using a membrane (MWCO 50,000) with deionized water.

Preparation of suprastructures with GOX- and HRP-carrying NPs

The suprastructures were prepared by using aqueous dispersion of GOX-carrying NPs (60 mg/mL) and HRP-carrying NPs (60 mg/mL) mixture with a volume ratio of 1:2. To prepare the hemispherical suprastructure for 1.7 mm in diameter, the dispersion (5 μL) was dropped on the PDMS-grafted surface, followed by drying at room temperature for 1 h. The size of suprastructure was controlled by changing the volume of dispersion drops, e.g. 2.5, 5, 10, and 20 μL.

The spherical suprastructure was prepared by drying the same dispersion drop (5 μL) on the superamphiphobic surface. The superamphophobic surface was prepared by using the soot-templated method.[S2] Due to air cushion of the porous superamphiphobic surface, the dispersion drop was dried keeping its spherical shape, which generated spherical suprastructure after the evaporation process.

The porosity of the suprastructure was varied by using Ouzo drying effect.[S3] To prepare the Ouzo dispersion drop, ethanol (145 μL) and *trans*-anethole oil (5.3 μL) were added to a mixture of GOX- and HRP-NPs aqueous dispersion (100 μL). The mixture of 5 μL was dropped on the PDMS-grafted surface and dried for overnight. The suprastructure was washed out using ethanol to remove the oil. After removing the oil, micropores were formed inside the suprastructure, which increased the porosity.

Characterization of reaction kinetics of suprastructures

Different concentrations of glucose solution (0, 1.2 μM, 0.78, 3.1, 12.5, 50, and 200 mM) containing amplex red (50 μM) was dropped onto each suprastructure to initiate the reactions. The supernatant (1 μL) was taken at the interval of 30 s, and totally monitored for 180 s. Fluorescence was measured at excitation 550 nm/emission 595 nm with 100 times dilluted solution (measurement volume: 100 μL).

Michaelis-Menten kinetics was obtained by taking the reaction velocity witin 150 s. V_{max} is anticipated saturation velocity of reaction. K_m is glucose concentration at the half V_{max}. k_{cat} is product amount (mol) divided by enzyme (GOX) amount (mol).

Enzymatic assay for GOX-NPs

The reaction cocktail was prepared using sodium phosphate buffer (900 µL, pH 7.4, 10 mM), Amplex red (2.5 µL, 1 mM) dissolved in DMSO and glucose solution (100 µL, 100 mM). To assay the GOX acivity, the reaction mixture (100 µL) was placed in a well of a 96-well plate, then 0.5 µL of GOX-NPs dispersion was added, and the changes in fluorescence at excitation 555 nm and emission 595 nm were monitored by a TECAN plate reader (Infinite® M1000, Männedorf, Switzerland).

Glucose assay using hemisphere suprastructure with GOX- and HRP-carrying NPs

To perform glucose assay using suprastructure and NP dispersion, KI (150 mM) solutions and respective glucose solutions (125 mM, 12.5 mM, and 1.25 mM in deionized water) were mixed together in 1:4 volume ratio as a reaction cocktail, then, dropped onto the suprastructures with 1.2 µL. For detection of lower glucose concentration (< 1 mM), Amplex red (24 µM) solutions and respective glucose solutions (50 µM, 10 µM, and 1 µM in deionized water) were mixed together in 1:9 volume ratio, then dropped onto the assemblies with 1.2 µL. Changes in color were observed by naked eyes and were taken by photo. The images were analyzed by ImageJ software. In addition, light (wavelength: 400 nm) absorbance of the sample solution drop was measured by a Nanodrop (ND8000, Thermo Scientific, Waltham, Massachusetts, United States).

To perform glucose assay using NP dispersion, KI (150 mM) solutions and respective glucose solutions (125 mM, 12.5 mM, and 1.25 mM in deionized water) were mixed together in 1:4 volume ratio as a reaction cocktail, then, added 1.2 µL of the mixture to the 5 µL of NP-dispersion. For detection of lower glucose concentration (< 1 mM), Amplex red (24 µM) solutions and respective glucose solutions (50 µM, 10 µM, and 1 µM in deionized water) were used.

Preparation of suprastructure using commercially available silica nanoparticles with GOX and HRP

Aqueous dispersion (1 mL) containing silica nanoparticle (25 mg) and enzyme (4 mg GOX and 1 mg HRP) was prepared. To prepare the hemisphere suprastructure, the dispersion (5 μL) was dropped on the PDMS-grafted surface, followed by dried at room temperature for 1 h. To perform glucose assay, KI (150 mM) solutions and respective glucose solutions (12.5 mM in deionized water) were mixed together in 1:4 volume ratio as a reaction cocktail, dropped onto the suprastructures with 1.2 μL, then reacted for 20 min. Changes in color were observed by naked eyes and were taken by photo.

Preparation of hemisphere suprastructures with GOX-carrying NPs and TiO_2-NPs

The suprastructures were prepared by using a mixture of GOX-carrying NPs (40 mg/mL) and TiO_2 NPs (40 mg/mL) with a volume ratio of 9:1. The dispersion (5 μL) was dropped on the PDMS grafted surface, followed by drying at room temperature for 1 h.

Glucose assay using hemisphere suprastructure of GOX-carrying NPs and TiO_2-NPs

To perform glucose assay, KI (150 mM) solutions and respective glucose solutions (125 mM, 12.5 mM, and 1.25 mM in deionized water) were mixed together in 1:4 volume ratio, then, dropped onto the suprastructures with 1.2 μL. The GOX reactions with glucose were allowed for 9 min, then, UV was irradiated in the UV chamber to the reactant for 1 min (light source: 11W UV-A). Changes in color were observed by naked eyes and were taken by photo. The images were analyzed by ImageJ software.

Glucose assay from human urine

Initial glucose concentration in plain urine was measured by a glucometer (MedNet GmbH, Germany). To prepare a diabetic level of urine, the glucose solution was further added to the plain urine. The

suprastructure were prepared by using GOX-carrying NPs and HRP-carrying NPs with a volume ratio of 1:2. The dispersion was dried on the PDMS graft surface. To perform glucose assay, urine and KI (300 mM) solution were mixed together in 9:1 volume ratio, then, dropped onto the suprastructures with 1.5 µL. Changes in color were observed by naked eyes and were taken by photo. After 20 min, light (wavelength: 400 nm) absorbance of the sample solution drop was measured by a Nanodrop for estimating glucose concentration.

Glucose assay from human serum

All experiments using human serum were performed in compliance with the relevant laws and institutional guidelines. The institutional ethics committee had approved the study (Landesärztekammer Rheinland-Pfalz, 837.439.12 (8540-F)). Written informed consent was obtained for any experimentation with human subjects. Human blood was obtained from the Department of Transfusion Medicine in Universitätsmedizin Mainz from healthy donors in accordance with the Declaration of Helsinki.

Blood was clotted overnight according to the standard protocol to generate human serum. A serum pool from ten volunteers was used and storage at -80 °C. To remove protein aggregates after thawing, human serum was centrifuged for 30 min at 20,000 G before usage. Initial glucose concentration in plain serum was measured by a glucometer (MedNet GmbH, Germany). To prepare a diabetic level of serum, the glucose solution was further added to the plain serum. The suprastructure were prepared by using GOX-carrying NPs and HRP-carrying NPs with a volume ratio of 1:2. The dispersion was dried on the PDMS graft surface. To perform glucose assay, serum and amplex red (10 mM) solution were mixed together in 9:1 volume ratio, then, dropped onto the suprastructures with 1.5 µL. After reaction for 20 min, the reactant of 1 µL was added to phosphate buffer (pH 7.4) of 100 µL in a 96-well plate. Changes in fluorecence were monitored by platereader (excitation 555 nm, emission 595 nm).

Table S1. Static contact angle (CA) and dynamic contact angles of water drops on the PDMS-grafted surfaces. Each value was averaged with 5 tested results.

Liquid	Static CA [°]	Advancing CA [°]	Receding CA [°]	CA hysteresis [°]
Deionized water	105 ± 0.3	107 ± 0.2	100 ± 0.2	7 ± 0.3
Nanoparticles aqueous dispersion	102 ± 0.1	108 ± 0.9	87 ± 0.3	21 ± 1.1

Table S2. Characteristics of GOX-, HRP-, and TiO_2-NPs.

	GOX -NPs[***]	HRP-NPs[***]	TiO_2-NPs (P25) **

Diameter (nm)	191[*]	184[*]	25[**]
Zeta potential (mV)	-30 ± 8	-35 ± 4	-39 ± 1
Average pore size (nm)	4.7	4.7	N/D
Pore volume (cm^3/g)	0.11 ± 0.01[***]	0.16 ± 0.04 [***]	N/D
Density (g/cm^3)	1.99 [***]	1.90	4.23**

[*]Measured by dynamic light scattering, [**]Provided from the manufacturer

[***]S.-M. Jo, F. R. Wurm, K. Landfester, *ACS Appl. Mater. Interf.* 2018, 10, 34230

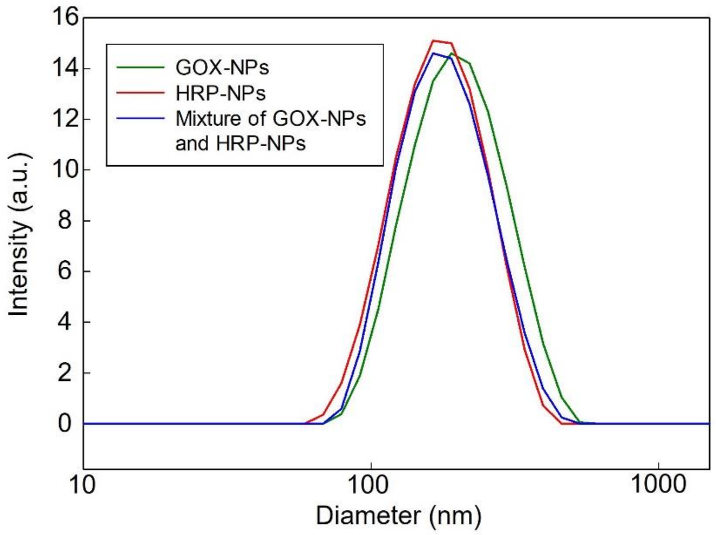

Figure S1. Size distribution of nanoparticles (NPs) dispersions: the GOX-NPs dispersion (black line), the HRP-NPs dispersion (red line), and the mixture dispersion of GOX- and HRP-NPs (blue line).

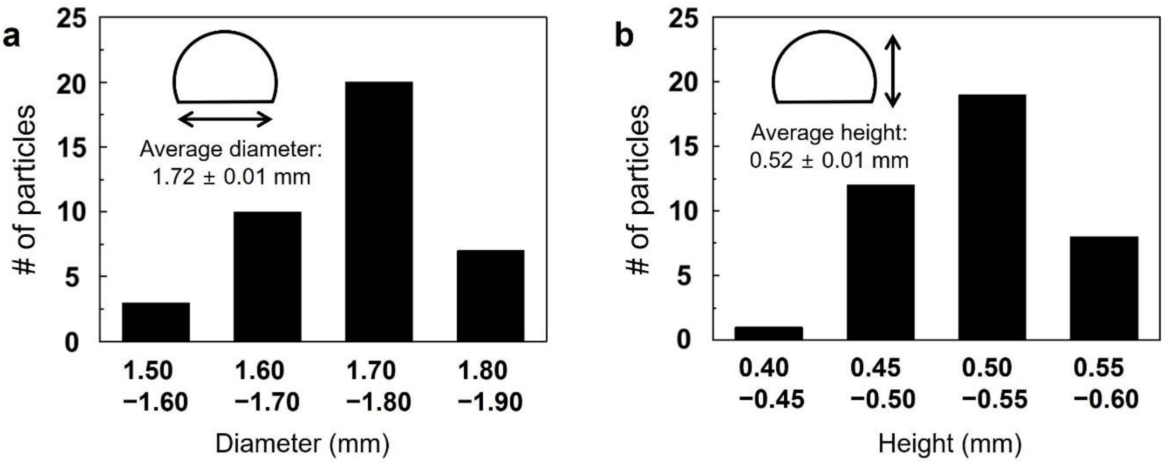

Figure S2. Size distribution of the suprastructures. (**a**) diameters and (**b**) heights of forty suprastructures were analyzed from optical microscope images of suprastructures.

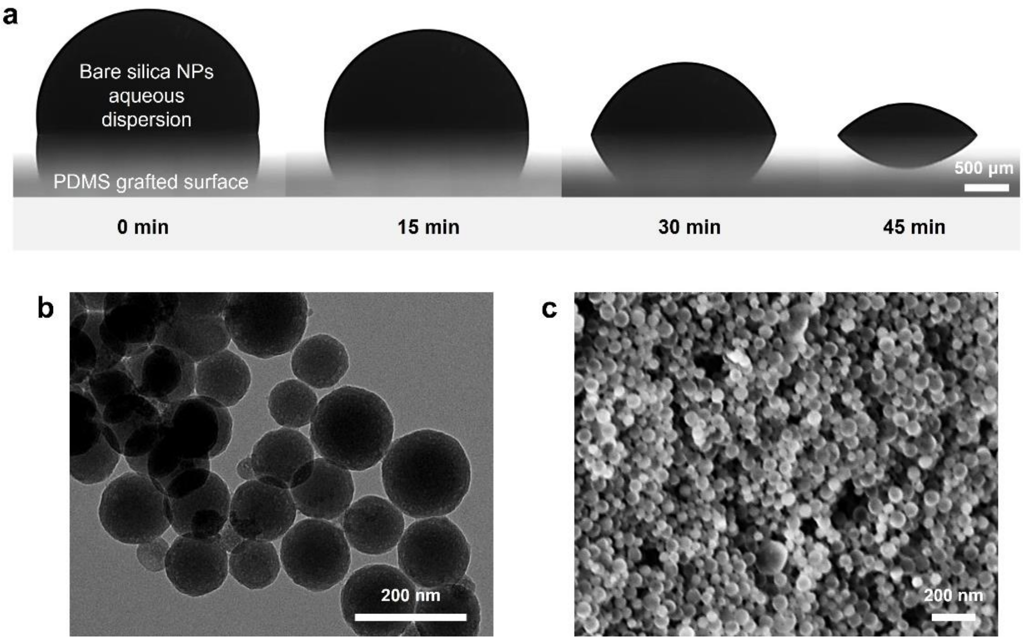

Figure S3. (**a**) Snapshot images of preparation of suprastructures without carrying enzymes via the S-TED method. (**b**) Transmission electron microscope (TEM) image of the bare silica nanoparticles (NPs) used for the suprastructure synthesis. (**b**) Scanning electron microscope (SEM) image of the assembled structure of bare silica NPs on the surface of the suprastructure.

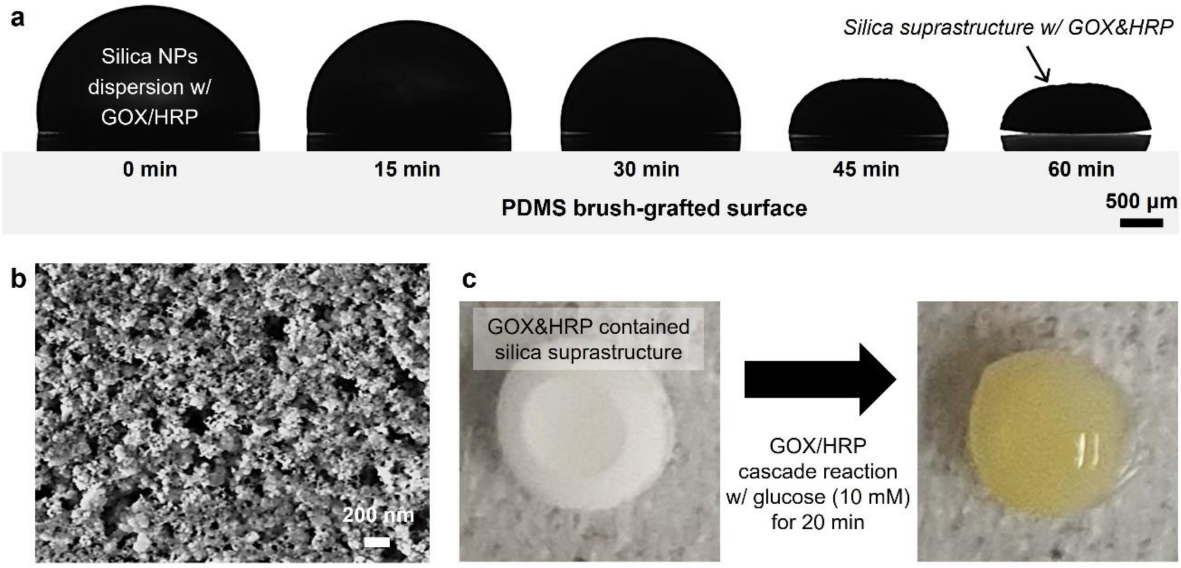

Figure S4. Preparation of suprastructures by different kinds of silica nanoparticles. (**a**) Evaporation progress for the preparation of the GOX/HRP contained silica suprastructures by using commerially available silica NPs. (**b**) SEM image of the suprastructures. (**c**) Photographic pictures of the suprastructure (left) and the same suprastructure after enzymatic reaction with 10 mM glucose solution for 20 min (right).

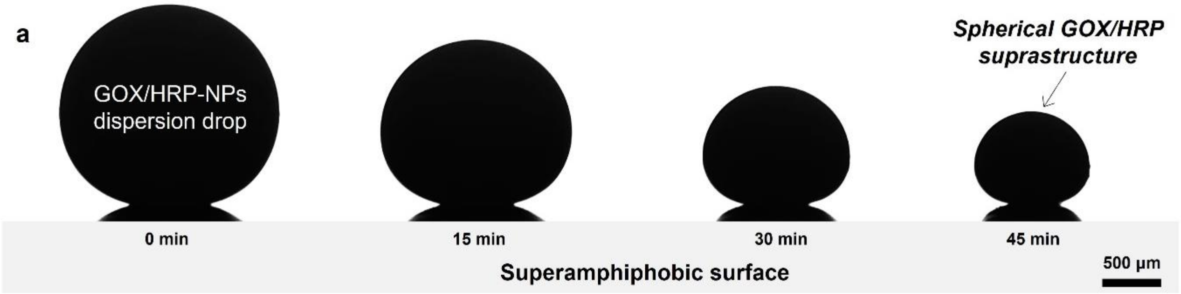

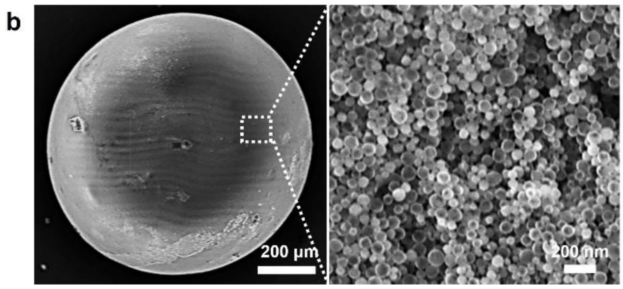

c

Michaelis-Menten kinetics	
K_m (mM)	16.3
V_{max} (pmole s^{-1})	0.41
k_{cat} (s^{-1})	5.4 x 10^{-3}
k_{cat}/K_m (mM^{-1} s^{-1})	3.3 x 10^{-4}

Figure S5. Characteristics of spherical suprastructures. (**a**) Evaporation progress for the preparation of the spherical GOX/HRP suprastructures on superamphiphobic surface. The superamphiphobic surface was prepared by the soot-templated method.[S2] (**b**) SEM images of the spherical suprastructure. The right image is a high magnification image of the surface of the suprastructure. (**c**) Michaelis-Menten kinetics (reaction kinetics by Michaelis-Menten plot) of the spherical suprastructures in terms of GOX/HRP cascade reaction.

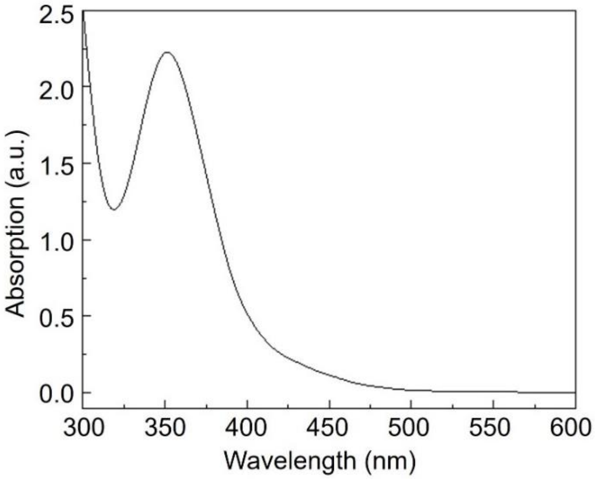

Figure S6. UV-visible spectra of aqueous I_2 solution in water.

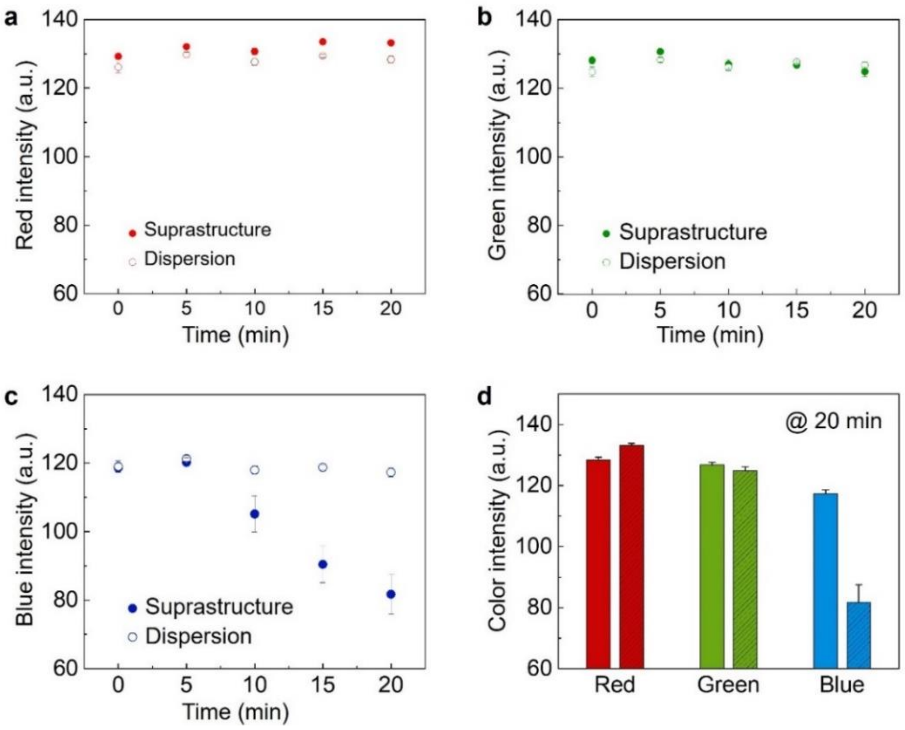

Figure S7. Changes in RGB elements ((**a**) red, (**b**) green, and (**c**) blue) of enzyme-carrying NPs dispersion drop and enzyme-carrying suprastructure after the enzymatic reactions with 10 mM glucose. (**d**) The color intensities of enzyme-carrying NPs dispersion drop (left bars) and enzyme-carrying suprastructure (right bars,diagonal checked) after 20 min enzymatic reactions.

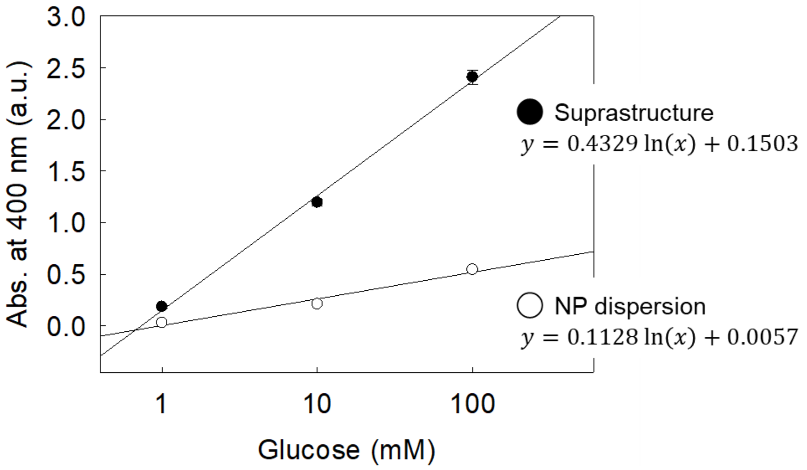

Figure S8. A standard curve of GOX/HRP cascade reactions for respective glucose concentrations measured from Figure 2c. The absorbance at 400 nm was measured after 20 min reaction.

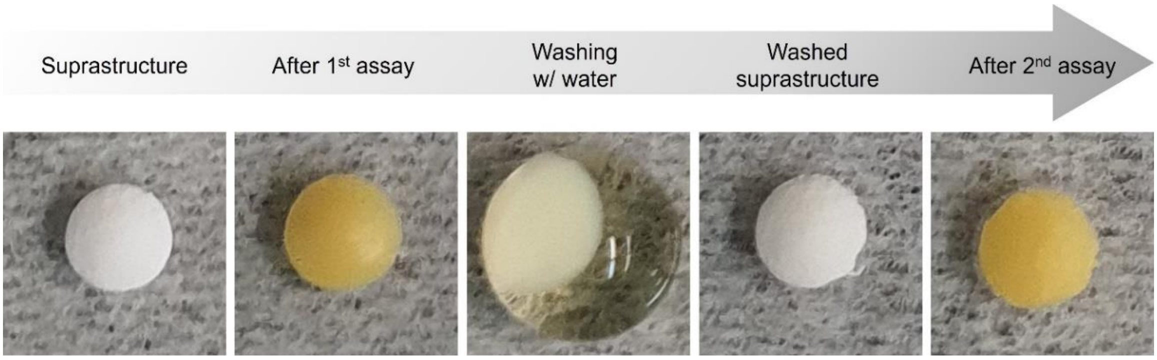

Figure S9. Pictures of multiple enzymatic assays by using GOX-suprastructure. After the first assay with 10 mM glucose drops for 20 min reaction (the second left image), the remaining reactants and products (glucose, KI, and I_2) were washed with water (the middle image). Then, the glucose assay was carried out on the same suprastructure (the last image).

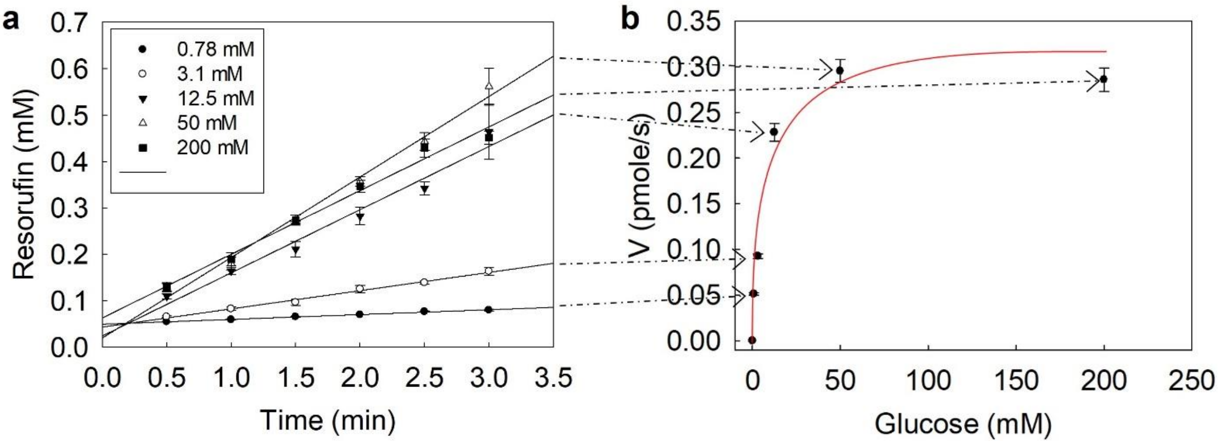

Figure S10. Reaction kinetics (V_{max} and K_m) of enzymatic cascade reaction of hemispherical suprastructure. (**a**) Concentration variations of resorufin as enzymatic cascade reaction with different glucose concentrations (0.78, 3.1, 12.5, 50, and 200 mM). The resorufin was produced from Amplex red dye converted by the enzymatic cascade reaction of the suprastructure. The concentrations of resorufin were characterized by Nanodrop measurement, and the reaction velocities (V) at different glucose concentrations were analyzed by fitting the slopes. (**b**) Michaelis-Menten plot obtained by the reaction velocities. The V_{max} and K_m were calculated from the Lineweaver-Burk plot consisting of reciprocal values of the Michaelis-Menten plot.

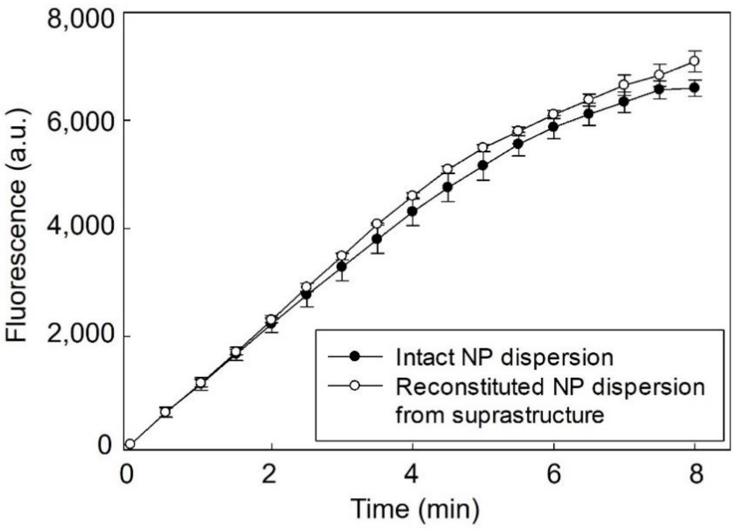

Figure S11. Enzymatic activities of NPs dispersions. Intact NPs dispersion indicates the mixed dispersion of GOX- and HRP-NPs before preparation of the superstructure. Reconstituted dispersion indicates the NPs dispersion obtained by redispersing the suprastructure in water. The assay was performed using Amplex red as a dye, measuring fluorescence at excitation 555 nm/emission 595 nm. After 8 min, the values are saturated.

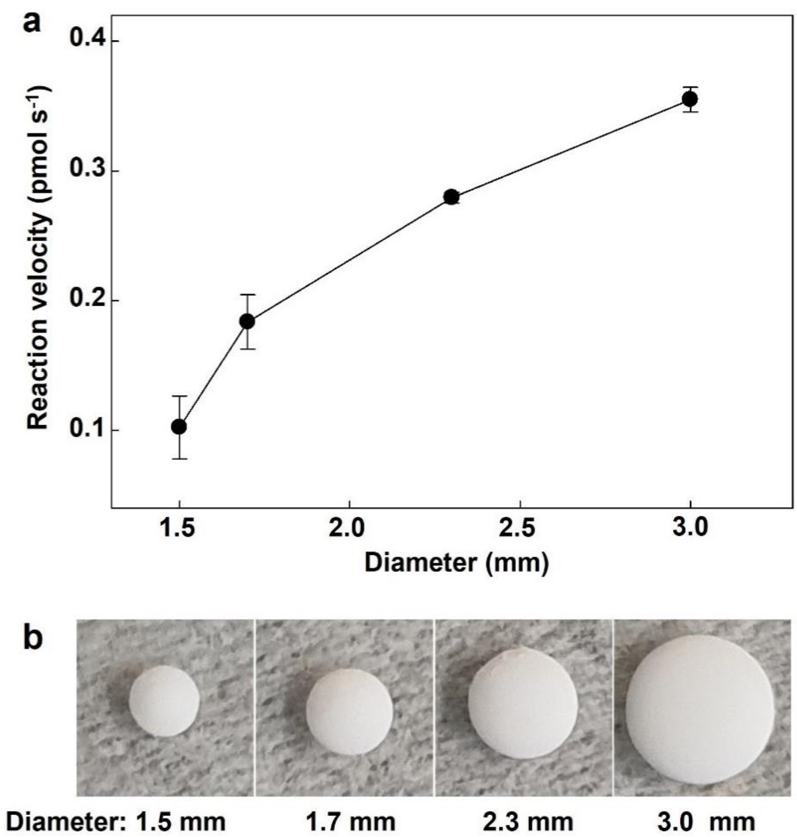

Figure S12. Effect of size of suprastructures on enzymatic reaction kinetics. The reaction velocities of suprastructures were obtained by the reaction with 10 mM glucose. Diameters of 1.5, 1.7, 2.3, and 3.0 mm suprastructures were prepared by different volumes of GOX- and HRP-NPs dispersion (1 vol%), 2.5, 5, 10, 20 µL, respectively.

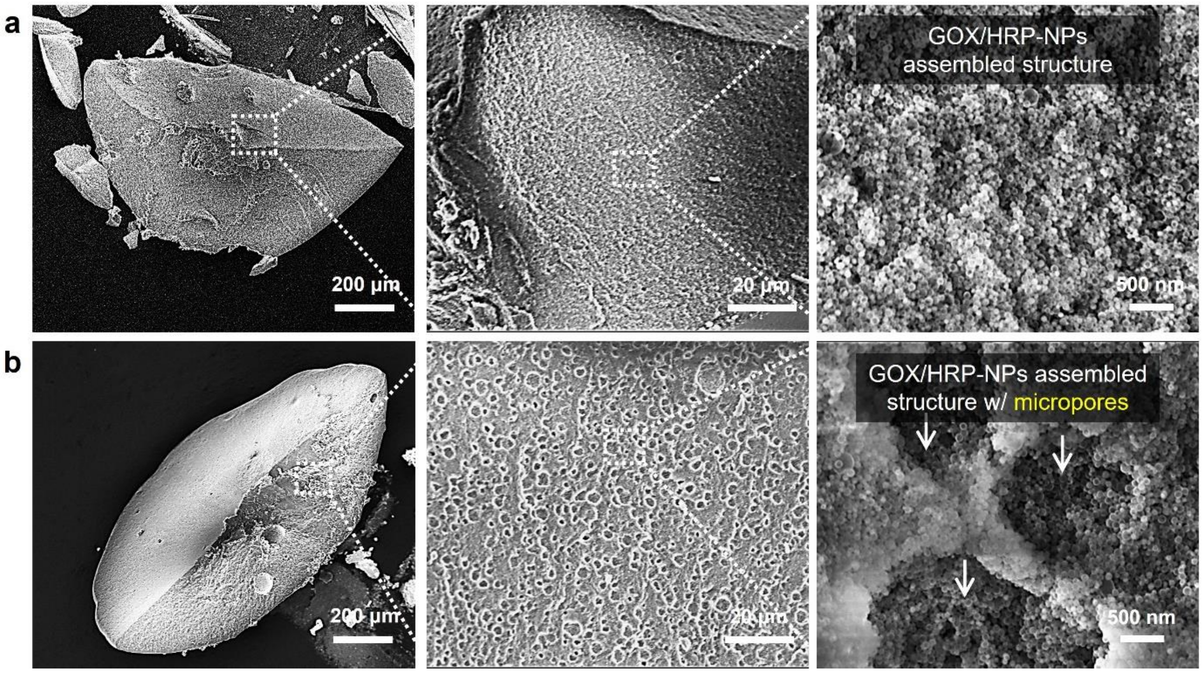

Figure S13. Scanning electron microscope (SEM) images of suprastructures having different porosities of (**a**) 68 ± 0.7 and (**b**) 75 ± 0.4 %, prepared by the GOX/HRP-NPs aqueous dispersion and the GOX/HRP-NPs Ouzo dispersion, respectively. The Ouzo dispersion includes trans oil (~2 wt%), which produces micropores after evaporation. Due to the micropores (indicated by white arrows in the last image of b)), the porosity of the suprastructure fabricated from the Ouzo dispersion is higher than that fabricated from the aqueous dispersion.[S3]

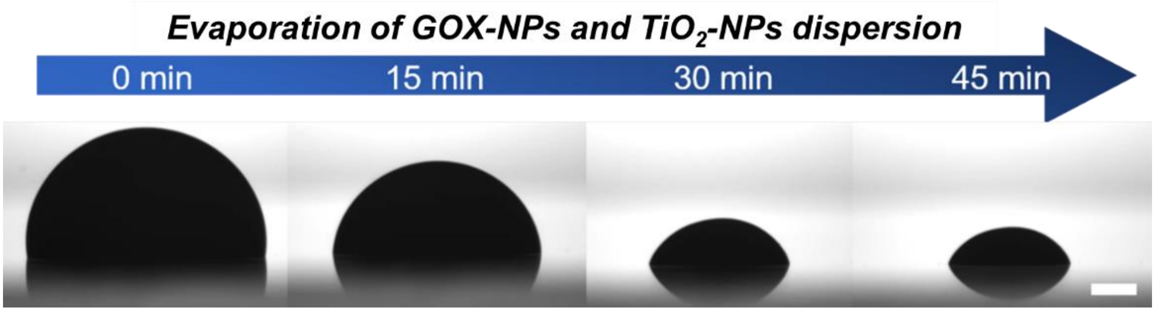

Figure S14. Evaporation progress for the fabrication of GOX- and TiO_2-carrying suprastructure on the PDMS grafted surface. Scale bar indicates 500 μm.

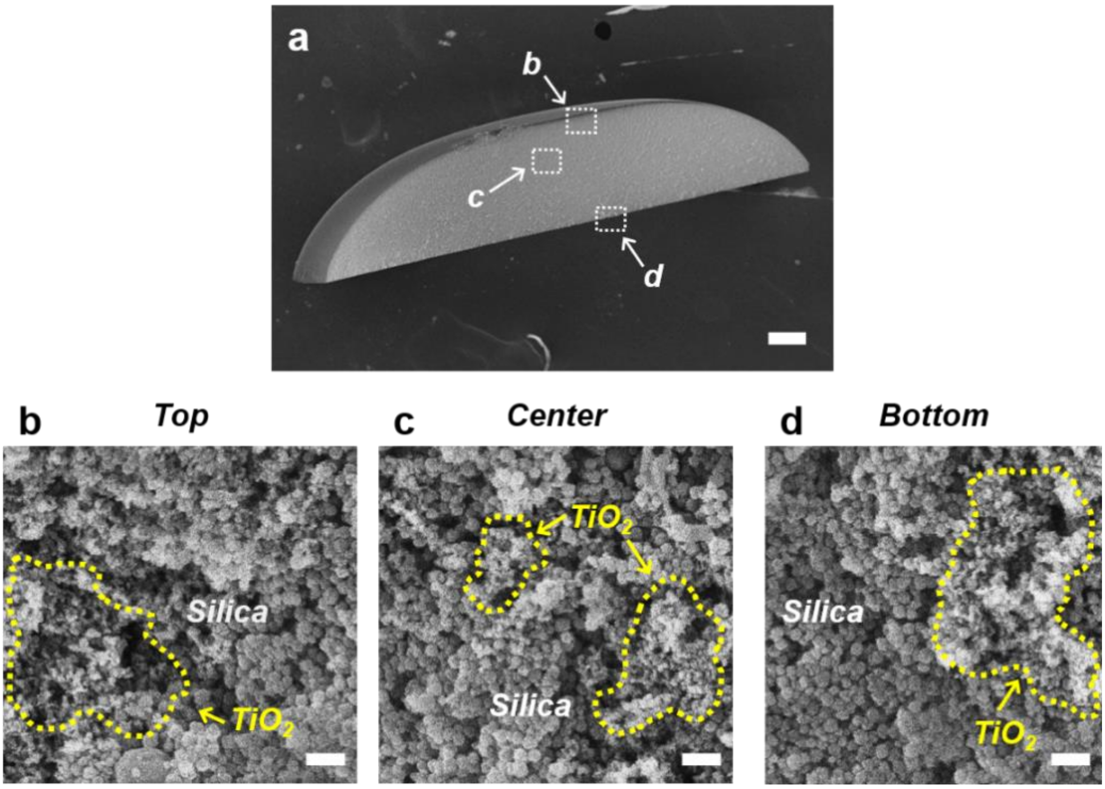

Figure S15. Scanning electron microscope (SEM) images of (**a**) cross-section of GOX- and TiO_2-carrying suprastructures, and high magnification images of the (**b**) top, (**c**) center, and (**d**) bottom of the suprastructures, respectively. Scale bars indicate 100 μm for (**a**) and 300 nm for (**b-d**), respectively.

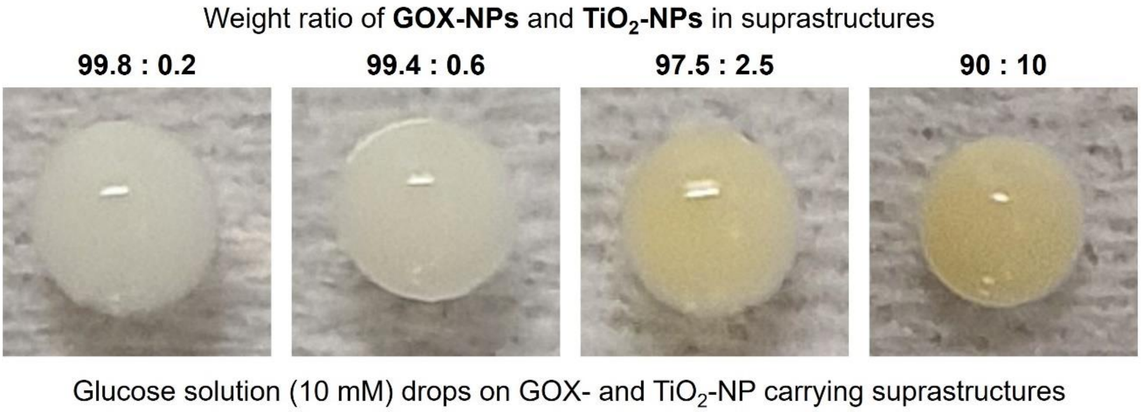

Figure S16. Photographic pictures of glucose solution drops on GOX/TiO_2-carrying suprastructures consisting of different ratios of GOX and TiO_2. After dropping the glucose solution, the reaction of GOX with glucose (10 mM) was allowed for 9 min, then UV-A was irradiated for 1 min to active TiO_2 photocatalytic reactions. KI was used as the indicator, therefore, the yellow-brown color indicates the progression of the reactions.

References

[S1] S.-M. Jo, F. R. Wurm, K. Landfester, *ACS Appl. Mater. Interf.* **2018**, 10, 34230

[S2] X. Deng, L. Mammen, H.-J. Butt, D. Vollmer, *Science* **2012**, 335, 67

[S3] H. Tan, S. Wooh, H.-J. Butt, X. Zhang, D. Lohse, *Nat. Commun.* **2019**, 10, 478
